# Effect of preoperative pulmonary hemodynamic and cardiopulmonary bypass on lung function in children with congenital heart disease

**DOI:** 10.1007/s00431-023-04926-0

**Published:** 2023-03-18

**Authors:** Manuela Simonato, Massimo Padalino, Luca Vedovelli, Cristiana Carollo, Anna Sartori, Vladimiro Vida, Dario Gregori, Virgilio Carnielli, Paola Cogo

**Affiliations:** 1 Department of Women’s and Children’s Health, University of Padova, Corso Stati Uniti 4, 35127 Padua, Italy; 2 PCare Laboratory, Fondazione Istituto Di Ricerca Pediatrica, “Città Della Speranza”, Padua, Italy; 3 Pediatric and Congenital Cardiac Surgical Unit, Department of Cardiac, Thoracic and Vascular Sciences, University of Padova, Padova, Italy; 4 Unit of Biostatistics, Epidemiology and Public Health, Department of Cardiac, Thoracic, Vascular Sciences and Public Health, University of Padova, Padua, Italy; 5 Anesthesiology and Intensive Care Unit, Department of Medicine-DIMED, University of Padova, Padua, Italy; 6 Division of Neonatology, Polytechnic University of Marche and “G. Salesi” Children’s Hospital, Ancona, Italy; 7 Department of Medicine, University Hospital S. Maria Della Misericordia, University of Udine, Udine, Italy

**Keywords:** Children, Congenital heart disease, Cardiopulmonary bypass, Mechanical ventilation, Lung, Pulmonary blood flow

## Abstract

In children with congenital heart disease (CHD), pulmonary blood flow (Qp) contributes to alterations of pulmonary mechanics and gas exchange, while cardiopulmonary bypass (CPB) induces lung edema. We aimed to determine the effect of hemodynamics on lung function and lung epithelial lining fluid (ELF) biomarkers in biventricular CHD children undergoing CPB. CHD children were classified as high Qp (*n* = 43) and low Qp (*n* = 17), according to preoperative cardiac morphology and arterial oxygen saturation. We measured ELF surfactant protein B (SP-B) and myeloperoxidase activity (MPO) as indexes of lung inflammation and ELF albumin as index of alveolar capillary leak in tracheal aspirate (TA) samples collected before surgery and in 6 hourly intervals within 24 h after surgery. At the same time points, we recorded dynamic compliance and oxygenation index (OI). The same biomarkers were measured in TA samples collected from 16 infants with no cardiorespiratory diseases at the time of endotracheal intubation for elective surgery. Preoperative ELF biomarkers in CHD children were significantly increased than those found in controls. In the high Qp, ELF MPO and SP-B peaked 6 h after surgery and tended to decrease afterward, while they tended to increase within the first 24 h in the low Qp. ELF albumin peaked 6 h after surgery and decreased afterwards in both CHD groups. Dynamic compliance/kg and OI significantly improved after surgery only in the High Qp.

*  Conclusion:* In CHD children, lung mechanics, OI, and ELF biomarkers were significantly affected by CPB, according to the preoperative pulmonary hemodynamics.

**Table:** 

**What is Known:** *• Congenital heart disease children, before cardiopulmonary run, exhibit changes in respiratory mechanics, gas exchange, and lung inflammatory biomarkers that are related to the preoperative pulmonary hemodynamics.* *• Cardiopulmonary bypass induces alteration of lung function and epithelial lining fluid biomarkers according to preoperative hemodynamics.*	
**What is New:** *• Our findings can help to identify children with congenital heart disease at high risk of postoperative lung injury who may benefit of tailored intensive care strategies, such as non-invasive ventilation techniques, fluid management, and anti-inflammatory drugs that can improve cardiopulmonary interaction in the perioperative period.*

**What is Known:**

*• Congenital heart disease children, before cardiopulmonary run, exhibit changes in respiratory mechanics, gas exchange, and lung inflammatory biomarkers that are related to the preoperative pulmonary hemodynamics.*

*• Cardiopulmonary bypass induces alteration of lung function and epithelial lining fluid biomarkers according to preoperative hemodynamics.*

**What is New:**

*• Our findings can help to identify children with congenital heart disease at high risk of postoperative lung injury who may benefit of tailored intensive care strategies, such as non-invasive ventilation techniques, fluid management, and anti-inflammatory drugs that can improve cardiopulmonary interaction in the perioperative period.*

## Introduction

Congenital heart diseases (CHD) are associated with restrictive, obstructive, or diffusion abnormalities of the lungs, because of adverse changes in pulmonary hemodynamics occurring perinatally [1]. Increased pulmonary blood flow (high Qp) is associated with pulmonary edema and decreased pulmonary compliance, which improves significantly after surgical repair [2]. Lungs with low Qp are also compromised because of decreased lung volume [3, 4] and loss of the stabilizing effects of normal pulmonary hemodynamics on airways mechanics [5]. All these alterations profoundly affect the perioperative hemodynamics and intensive care management as well as they have clinical consequences in the perioperative and long-term outcomes [6].

Notably, while cardiac surgical repair improves lung mechanics in the high Qp CHD, less is known about the effect of surgical correction in the low Qp CHD [7–10]. Two major competing factors determine the perioperative changes in lung function: on one side, the postoperative normalization of pulmonary blood flow is likely to improve the pulmonary mechanical properties [9], whereas on the other side, the intraoperative management including CPB, hypothermia, and positive pressure ventilation can induce lung edema [11, 12]. As a result, some authors reported a decrease in total respiratory resistance and an increase in compliance after cardiac surgery [7, 9], whereas others observed impairments in the resistive and elastic properties of the respiratory system [8]. There is no study assessing the role of Qp before surgery on the postoperative lung function and gas exchange in CHD requiring CPB for surgical correction.

Biomarkers of lung injury can help to better define the cardiopulmonary interaction during the perioperative period. Blind bronchoalveolar lavage (BAL) represents a potential method for describing in vivo changes on lung epithelial lining fluid (ELF) composition. Although BAL by elective fibro-optic bronchoscopy for research is ethically unacceptable in children, non-bronchoscopic tracheal aspirates (TA) are feasible when access to the lower airways is provided through an endotracheal tube, placed for clinical indications [13–15]. To estimate the TA dilution due to the sampling technique, the ratio between TA and plasma urea concentrations is used to normalize the TA components for the ELF [16].

We previously reported that CHD morphology affects the ELF composition [14], but nothing is known about their trend in the early postoperative period.

In this study, we sought to identify how CHD phenotype affects lung function and ELF biomarkers before and soon after CPB cardiac surgery and also to assess if these findings contribute to alterations of, lung mechanics, gas exchange and, as a consequence, to the need of respiratory support.

## Methods

### Subjects and protocol

We prospectively studied children with complex biventricular CHD undergoing surgical repair at our center between January 2018 and December 2020.

We enrolled children with large septal defects, right ventricular outflow tract obstructions (RVOTO), conal abnormalities, and anomalous venous return. All children were scheduled to elective cardiac surgery, with CPB time > 60 min, on stable hemodynamic condition not requiring inotropic support or fluid boluses before surgery.

Exclusion criteria were the need for mechanical ventilation before surgery, preexisting lung diseases, univentricular heart physiology, liver or renal failure (factor V < 20% and/or creatinine clearance < 30%), chromosomal abnormalities, or genetic diseases. Infants who required extracorporeal membrane oxygenation or open chest after surgery were also excluded.

All infants received a preoperative cardiological evaluation including electrocardiogram and 2D echocardiography. Cardiac catheterization was performed only if clinically indicated. All cardiological data were prospectively recorded in a dedicated database. We defined “low Qp” infants presenting with RVOTO associated with SaO_2_ < 90% in the perinatal period or with episodes of blue spells or cyanosis reported by the care givers or recorded by the attending cardiologist in infants waiting for surgery. Infants with ventricular septal defect, AV canal, without RVOTO, non-obstructed anomalous venous return, and transposition of the great arteries were considered “high Qp” infants.

Management of anesthesia was uniform in all children and was described elsewhere [17].

After administration of heparin with activated clotting time of about 400 s, CPB was initiated. During CPB, mild to deep hypothermia was applied, and lungs were not ventilated. Hypothermia during CPB was defined as mild (35.0–30.1 °C), moderate (30.0–25.1 °C), or deep (25.0–15.1 °C) [18]. A hematic prime was used to maintain hematocrit between 25 and 30% during CPB. Cold-blood cardioplegia was given every 20-min intervals during periods of aortic cross-clamping. Acid–base management was maintained as alpha-stat strategy until rewarming with a hematocrit goal of 30% during CPB. After rewarming, cross clamp was removed, and ventilation was started with an inspired oxygen fraction (FiO_2_) of 1.0. After modified ultrafiltration, the chest was closed, and the patient was transferred to the cardiac intensive care unit (CICU). Peri- and postoperative data were recorded until hospital discharge. Lung function was assessed by oxygenation index (OI) [19], and dynamic compliance calculated preoperatively, within 1 h from the arrival in the CICU and then at 6, 12, and 24 h after surgery. The dynamic compliance was determined using the equation: Cdyn = VT / (PIP − PEEP) where VT is the tidal volume, PIP is the peak inspiratory pressure, and PEEP is the positive end-expiratory pressure. Three dynamic compliance measurements were collected at each time point, and the results were averaged.

As controls, we collected a group of infants who underwent surgery on general anesthesia, with no history of chronic respiratory symptoms or recent upper or lower respiratory tract infections.

The study was approved by the local ethics committee (Protocol number 3142/AO/14 Padova Hospital, Padova, Italy), and parental written informed consent was obtained for all study patients.

TAs were collected in CHD and controls immediately after anesthesia induction and intubation, while in children with CHD, TAs were collected also at the end of surgery and 6, 12, and 24 h after CICU admission. Part of the fluid recovered from TA was used as such for MPO determination, and part was centrifuged at 400 × *g* for 10 min to sediment cells and cell debris. Aliquots of whole TAs and supernatants were stored at − 80 °C. At the same time point, 0.5 ml of fresh blood was drawn into vacutainer tubes containing ethylenediaminetetraacetic acid. After being centrifuged at 1400 × *g* for 10 min, the plasma was aliquoted and frozen at − 80 °C until analysis.

### Measurements

SP-B concentration was determined by ELISA [20, 21]. Albumin concentration was measured with the bromocresol green method [22]. MPO activity was measured as previously reported [23].

TA dilution was calculated by analyzing plasma and TA urea levels using a commercial kit (QuantiChrom urea assay kit, Bioassay system, Hayward, CA). TA samples with urea concentration below 0.08 mg/dL (linear detection range of the kit 0.08–100 mg/dL) were discarded. The ratio of plasma urea to TA urea was used to calculate the dilution of ELF, as previously described [16]. This dilution factor was applied to all TA biomarkers to obtain the ELF concentration.

### Data analysis

Variables were reported as median (interquartile range). To compare groups, we used the Mann–Whitney test. For intra-group comparison, we used the Kruskal–Wallis test with the Dunn’s as post hoc test and Holm multiple comparisons correction. Preoperative OI values were not included in the analysis because they are not reliable as indexes of oxygen diffusion capacity in the context of right to left shunt.

All tests were two-sided, and a *p*-value lower than 0.05 was considered statistically significant. Graphs were generated in R with the package “GGstatsplot” [24]. Statistical analysis was performed using R 4.1.2 [25]. All the code is freely available at https://osf.io/hye98/.

## Results

### Population description

Sixty children with biventricular CHD undergoing elective cardiac surgery were included. Six infants (10%) were born preterm, and 12 (20%) were neonates. The median age at cardiac surgery was 3.7 (1.7; 5.5) months, 47% were male, and the median weight was 5.5 (3.8; 6.3) kg. Median surgery and CPB times were 223 (190; 245) and 120 (97; 143) min, respectively. Median length of mechanical ventilation and CICU stay were 2.0 (1.3; 3.0) and 4.0 (2.5; 5.6) days, respectively. One infant (1.7%) died before hospital discharge.

Forty-three infants belonged to the high Qp, and 17 to the low Qp groups; their baseline clinical and surgical characteristics are reported in Table 1. Preoperative age and weight, O_2_ saturation, surgery and CPB time, and duration of hypothermia were significantly different between the two groups. Three children were extubated before the end of the study (24 h post-surgery), all belonging to the high Qp group.

**Table 1:** Baseline characteristics and operative variables of the study groups

	High Qp (*N* = 43)	Low Qp (*N* = 17)	*p*^a^
**Baseline characteristics**			
Term/preterm	41/2	13/4	0.001
Gender (M/F)	20/23	8/9	0.483
Age at surgery (months)	3.0 (0.9; 4.3)	5.6 (4.0; 6.7)	< 0.0001
Weight (kg)	4.2 (3.5; 6.0)	6.3 (5.4; 7.3)	< 0.0001
SaO_2_ (%)	97 (93; 100)	89 (77; 92)	< 0.0001
**Diagnosis (N°)**			
TGA-IVS	10		
TOF	8	14	
VSD/AVC	21	3	
TAPV	4		
**Surgical characteristics**			
Surgery time (min)	205 (180; 239)	240 (210; 260)	0.0160
CPB time (min)	109 (74; 130)	138 (117; 150)	0.0042
Hypothermia time (min)	70 (50; 88)	100 (77; 120)	0.0003
Rewarming time (min)	20 (15; 30)	30 (20; 37)	0.2290
Minimum temperature (°C)	32.0 (30.2; 33.5)	32.0 (31.2; 32.0)	0.8655
Minimum temperature time (min)	40 (30; 50)	50 (20; 60)	0.3819
MV time (d)	2.2 (1.3; 4.0)	2.0 (1.2; 2.6)	0.5676
CICU time (d)	3.7 (2.6; 5.6)	4.0 (2.0; 5.6)	0.9168

High Qp: increased pulmonary blood flow, Low Qp: decreased pulmonary blood flow, SaO_2_: arterial oxygen saturation, *TGA-IVS* Transposition of Great Arteries with Intact Ventricular Septum, *TOF* Tetralogy of Fallot, *VSD* Ventricular Septal Defect, *TAVP *Total Anomalous Pulmonary Venous Drainage, *CPB* Cardiopulmonary Bypass, *MV* Mechanical Ventilation, *CICU* Cardiac Intensive Care Unit

^a^Exact *p* value by Mann–Whitney test

Sixteen infants served as controls, and their median age and weight at surgery were 0.7 (0.1–2.1) months and 3.1 (2.4–3.8) kg, respectively. They underwent elective surgery for anorectal (*n* = 5), facial (*n* = 1), and gastro-intestinal malformations (*n* = 4), esophageal atresia repair (*n* = 3), subdural hematoma (*n* = 1), retroperitoneal mass (*n* = 1), and central venous line placement (*n* = 1).

Among the TA samples collected during the study, we discarded one hematic sample belonging to the high Qp and 10 samples (6 in the high Qp and 4 in the low Qp) with low urea concentration in the TA (below 0.08 mg/dL, i.e., the sample was too diluted).

### Preoperative ELF markers

**Fig. 1:**
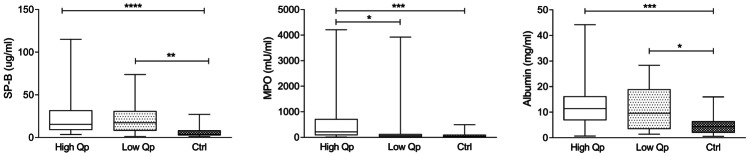
Preoperative epithelial lining fluid surfactant protein B (SP-B) and albumin concentration and myeloperoxidase activity (MPO) in the CHD groups (high Qp and low Qp) and in control infants (CTRL). Two asterisks (**) *p* < 0.01; three asterisks (***) *p* < 0.001 by Mann–Whitney test

As depicted in Fig. 1, high Qp and low Qp infants exhibited significantly increased ELF SP-B compared to controls (*p* < 0.0001 and *p* = 0.0011, respectively) before surgery. ELF MPO and albumin were significantly increased in the high Qp than in controls (MPO *p* = 0.0007 and albumin *p* = 0.0006, respectively), whereas only ELF albumin was significantly higher in the low Qp compared to controls (*p* = 0.0494). Preoperative ELF MPO was significantly higher in the high Qp compared to the low Qp groups (*p* = 0.0211).

### Intra- and postoperative lung function parameters and ELF markers

#### Intra group comparison

**Fig. 2:**
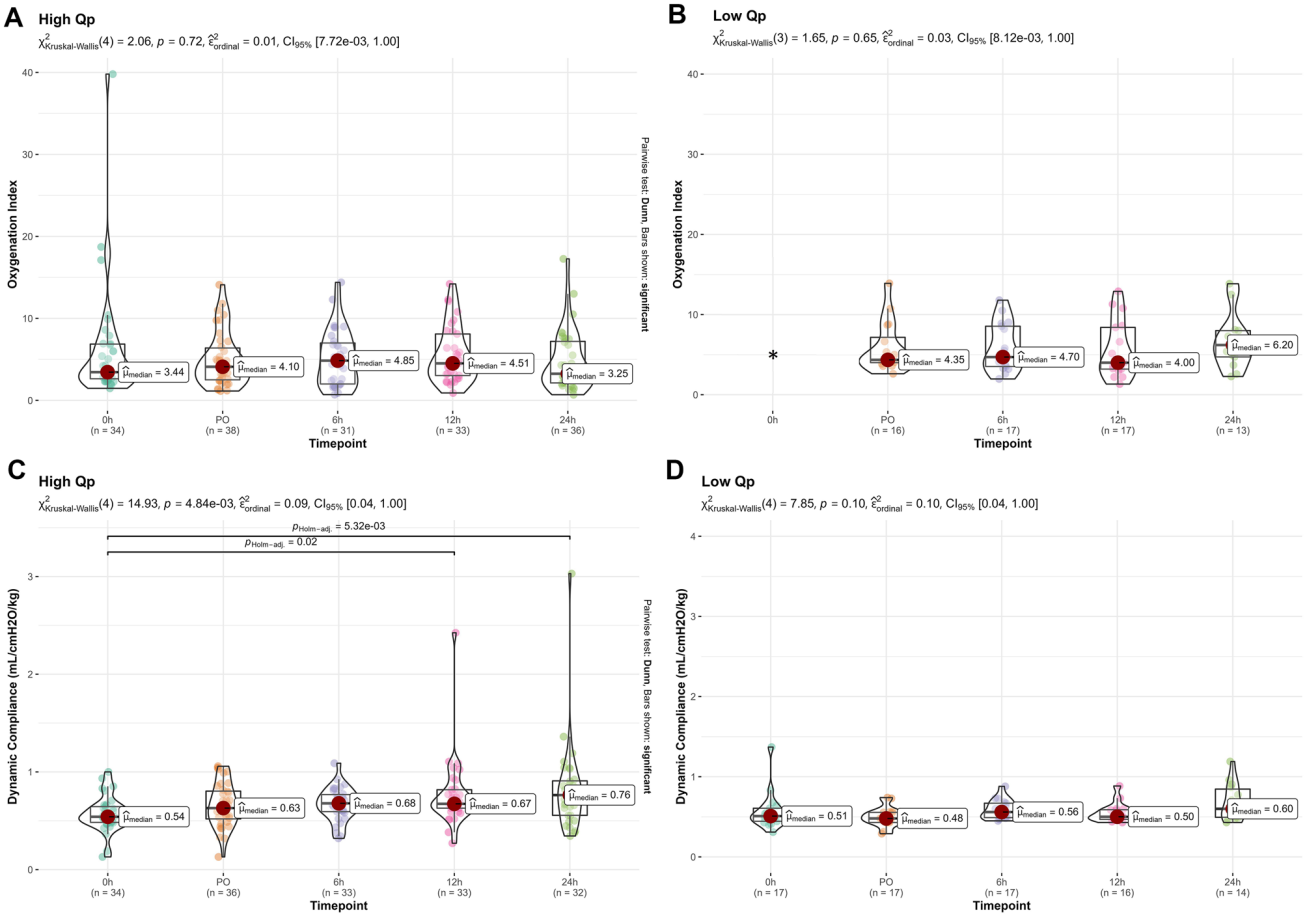
Oxygenation index and dynamic compliance trend over time in high and low Qp group. Data are represented as median and interquartile range. Kruskal–Wallis test for intra-group comparison with the Dunn’s as post-hoc test and Holm multiple comparisons correction. Asterisk (*), low Qp OI at time 0 is omitted because its value is not accurate in the presence of a right-to-left shunting that occurs in the CHD with right ventricular outflow obstruction (RVOTO)

In the high Qp group, the OI peaked 6 h after surgery to decrease to pre-surgery values after 24 h. In the low Qp group, OI reached the highest value at 24 h after surgery (Fig. 2, upper panel). Similarly, the dynamic compliance/kg improved significantly over the study period only in the high Qp group (*p* = 0.005), the values before surgery being significantly lower than those at 12 (*p* = 0.02) and at 24 h (*p* = 0.005) after surgery. In the low Qp group, dynamic compliance/kg did not improve over the study time (Fig. 2, lower panel).

**Fig. 3:**
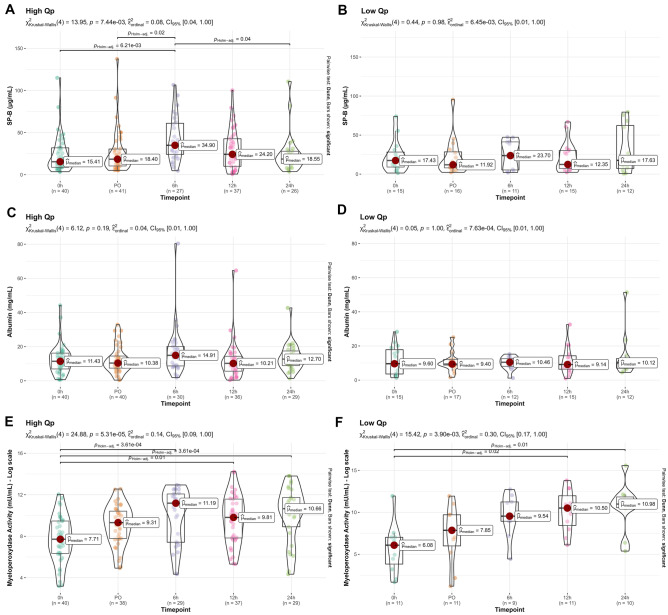
Surfactant protein B (SP-B) concentration, albumin concentration, and myeloperoxidase activity (MPO) trend over time in the high and low Qp groups. Data are represented as median and interquartile range. Kruskal–Wallis test for intra-group comparison with the Dunn’s as post hoc test and Holm multiple comparisons correction

ELF albumin values did not show any significant difference over time and reached the highest value 6 h after surgery in both CHD groups (Fig. 3, middle panel). ELF SP-B trend showed significant differences only in the high Qp group (*p* = 0.007) where it reached its maximum value 6 h after surgery to then decrease steadily in the first 24 h after surgery. In the low Qp, ELF SP-B concentrations fluctuated with no significant difference among the time points of the study (Fig. 3, upper panel). The ELF MPO significantly changed over time in both groups (high Qp *p* < 0.0001, low Qp *p* = 0.0061). In the high Qp group, the ELF MPO measured before surgery was significantly lower from that measured at 6 (*p* = 0.0004), 12 (*p* = 0.01), and 24 h (*p* = 0.0004) after surgery. In low Qp group, the ELF MPO measured before surgery was significantly lower from that measured at 12 (*p* = 0.02) and 24 h (*p* = 0.001) after the surgery (Fig. 3, lower panel).

#### Inter group comparison

The dynamic compliance/kg was significantly higher in the high Qp with respect to low Qp group at the end of the surgery (*p* = 0.009) and 12 h after surgery (*p* = 0.0004).

## Discussion

In this study, we demonstrated that CHD children undergoing CPB exhibited changes in respiratory mechanics, alveolar oxygen diffusion, and ELF lung biomarkers. These changes persisted during the first 24 h after surgery according to the preoperative hemodynamics. We evaluated ELF MPO activity and ELF SP-B concentrations since they are specific markers of acute lung inflammation, as previously reported [14, 26–28]. Also, we used ELF albumin concentration as marker of the alveolar-capillary leak. This has already been reported to be increased in CHD with high Qp, after CPB, and in preterm infants with respiratory distress syndrome [13–15].

### CHD lung function before and after surgery

Infants with CHD present since birth significant abnormalities of lung volumes, pulmonary elastic properties, and resistance to airflow, which may persist over time, and can significantly increase morbidity and affect quality of life during childhood and adulthood [29]. A large cohort of 834 children (555 CHD and 279 control children) showed that CHD children presented with significantly increased incidence of obstructive and restrictive patterns when compared to controls especially in complex cardiac anomalies, even without surgery [9]. When CHD children underwent one or two cardiac surgery procedures, the risk of having an impaired functional residual capacity of the lungs was increased of 5.4 and 12.9 folds, respectively. In addition, high Qp lungs were stiffer than normal, while the elasticity of the low Qp lungs was increased [8, 10, 30]. Lanteri et al. observed an improvement in lung compliances and resistances among children with increased Qp after cardiac surgery that were not detected in children with normal or decreased Qp [7].

Our study confirmed these observations by showing that in the high Qp, the dynamic compliance corrected for body weight was decreased before surgery and tended to improve over time, while in the low Qp group, it did not vary throughout the study period (Fig. 2). Similarly, OI improved over time in the high Qp, while it tended to become worse in the low Qp, suggesting the need for an increased mean airway pressure and FiO_2_ to maintain adequate gas exchange when the lungs are hypo-perfused before surgery. It is of note that indexes of oxygen diffusion capacity are not reliable in the preoperative phase of the low Qp group, because of the right-to-left shunting that occurs in the CHD with RVOTO.

The lung biomarkers mirrored the mechanical and oxygenation findings. Before surgery, ELF MPO, SP-B, and albumin were significantly higher in CHD infants compared to controls (Fig. 1). The high Qp group showed significantly increased level of ELF SP-B, MPO, and albumin, while the low Qp showed significantly higher ELF SP-B and albumin levels compared to controls suggesting that pulmonary blood overflow plays a central role in lung inflammation. This is also evident by the significantly higher ELF MPO found before surgery in the high Qp with respect to the low Qp group (Fig. 1).

After surgery, the markers of lung inflammation (ELF MPO, SP-B) peaked at 6 h in the high Qp group, while they steadily increased over the 24 h in low Qp. This finding suggests that lung inflammation lasts longer in CHD with the low Qp, which may contribute to a longer need of respiratory support after surgery. Unfortunately, positive pressure ventilation is reported to affect the hemodynamic stability of infants operated for RVOTO. Thus, alternative strategies to blunt the inflammatory reaction associated with CPB (i.e., anti-inflammatory drugs or exogenous surfactant) could be helpful to achieve a prompt spontaneous ventilation soon after cardiac surgery in these infants [31].

The ELF albumin was comparable in the two groups and did not change significantly over study time (Fig. 3). The leak of plasma albumin on the alveolar surface could play a role on endogenous surfactant inactivation, thus impairing alveolar stability and lung compliance. The albumin leak is cleared by alveolar macrophages that are reported to play a major role in resolution of tissue inflammation [32, 33].

Previous studies revealed different changing patterns in the mechanical parameters during the perioperative period in children with different CHD phenotypes, according to the Qp/Systemic blood flow (Qs) ratio [2, 10]. Impairments in airway and lung mechanics were observed in children with TOF after CPB, whereas aortic cross-clamping and chest closure were associated with marked decrease in the functional residual capacity and increased lung inhomogeneity in TOF and VSD [10]. These impairments were less severe in infants with ventricular septal defect (VSD) without RVOTO. The authors hypothesized that these mechanical deteriorations were probably caused by a diminished tethering effect of the lung periphery through a reduced filling of the pulmonary capillaries that seemed to be more pronounced in children with hypo-perfused lungs than in those with high Qp. Animal studies with restricted Qp supported this hypothesis [34]. The beneficial postoperative changes in lung mechanics occurring in the VSD infants were consequences of the reversal of the pulmonary vascular engorgement after surgical repair [10].

### Limitations

This study has some limitations. First, although the differences in ELF composition between the CHD infants and controls appear to be consistent, the wide variability of the biomarkers smoothed the differences between the two groups; in addition, we did not have samples collected at the end of the surgery for our control infants.

Secondly, the number of infants with low Qp is lower and they had longer CPB and rewarming time than those belonging of the high Qp group.

Furthermore, we did not make any correlation between the inflammatory process identified by the laboratory results and any imaging techniques, since we performed only chest X-ray postoperatively and not lung ultrasound that could have been a more sensitive technique to detect lung inflammation [35].

Samples obtained by tracheal aspiration could be also considered a limitation. However, TA composition, corrected by the dilution factor, is a widely accepted technique to represent the ELF composition of children unable to tolerate invasive procedures or in children where the bronchoscopy is not feasible for ethical reasons.

A significant strength of the present study lies in its longitudinal design, allowing sequential measurement of TA metabolites in a close interval of time.

## Conclusions

This study is the first which correlates the levels of inflammatory markers and albumin leak to lung mechanics and indexes of oxygen diffusion in CHD infants in the peri- and post-cardiac-operative period. Our findings could help to clarify the mechanism of respiratory failure after cardiac surgery, to device targeted ventilation and therapeutic strategies able to promote early extubation and spontaneous breathing [36].

This could be of paramount importance in CHD children with impending right ventricular failure, which requires spontaneous breathing or negative pressure ventilation to speed up the recovery of the right ventricular failure [31].

## Data Availability

The datasets generated during and/or analyzed during the current study are available from the corresponding author on reasonable request.

## Abbreviations

AVC: Atrioventricular canal
BAL: Bronchoalveolar lavage
Cdyn: Dynamic compliance
CHD: Congenital heart disease
CICU: Cardiac intensive care unit
CPB: Cardiopulmonary bypass
ELF: Epithelial lining fluid
FiO2: Inspired oxygen fraction
FRC: Functional residual capacity
MPO: Myeloperoxidase activity
OI: Oxygenation index
Qp: Pulmonary blood flow
Qs: Systemic blood flow
PEEP: Positive end-expiratory pressure
PIP: Peak inspiratory pressure
RVOTO: Right ventricular outflow tract obstruction
SaO2: Arterial oxygen saturation
SP-B: Surfactant protein B
TA: Tracheal aspirate
TOF: Tetralogy of Fallot
VSD: Ventricular septal defect
VT: Tidal volume
